# Coherence formation during narrative text processing: a comparison between auditory and audiovisual text presentation in 9- to 12-year-old children

**DOI:** 10.1007/s10339-020-01008-z

**Published:** 2021-01-06

**Authors:** Wienke Wannagat, Gesine Waizenegger, Gerhild Nieding

**Affiliations:** Department of Psychology, Developmental Psychology, University of Würzburg, Röntgenring 10, 97070 Würzburg, Germany

**Keywords:** Text comprehension, Multimodal narratives, Coherence, Children

## Abstract

In an experiment with 114 children aged 9–12 years, we compared the ability to establish local and global coherence of narrative texts between auditory and audiovisual (auditory text and pictures) presentation. The participants listened to a series of short narrative texts, in each of which a protagonist pursued a goal. Following each text, we collected the response time to a query word that was either associated with a near or a distant causal antecedent of the final sentence. Analysis of these response times indicated that audiovisual presentation has advantages over auditory presentation for accessing information relevant for establishing both local and global coherence, but there are indications that this effect may be slightly more pronounced for global coherence.

## Introduction

Children are exposed to texts daily from an early age. Narrative text is a very common text genre encountered by children. Bedtime stories are an important part of many families’ daily routine and throughout their time at elementary school, children become increasingly proficient and frequent readers. Many children’s books are illustrated, thus constituting visual narratives. Cognitive processing of visual narratives is a relatively new field of study (Cohn and Magliano 2020). While combinations of text and pictures have been researched extensively in the context of multimedia learning (e.g., Mayer 2005), far fewer studies have explored how visual narrative content affects the basic processes of establishing coherence, a central aspect of comprehension. Existing research on this matter has predominantly focused on adults’ processing of combinations of pictures and written language (e.g., Cohn and Wittenberg 2015; Magliano et al. 2016). The present study explores how pictures that illustrate auditory narrative text affect the processes of establishing coherence in children aged 9–12 years.

### Text comprehension and coherence formation

Text comprehension can be defined as a process of forming a coherent mental representation of the information conveyed by the text (Graesser et al. 1994; Kintsch 1988). This process involves at least three levels of representation (Dijk and Kintsch 1983): the first level is a quickly decaying representation of the text surface that represents the text information in its verbatim form. The second level, the text base, contains the text’s meaning usually represented as a network of coherently connected propositions. It serves as the foundation for the third level, the situation model that, as a representation of the whole situation described in the text, goes beyond the explicitly mentioned information by integrating the recipient’s prior knowledge via inference processes. Thus, coherence formation is a determining force during the construction of the text base and ultimately during text comprehension.

Coherence formation is determined by the limited working memory capacity and, therefore, is a sequential process that involves several processing cycles (Kintsch 1988). During each cycle, the information currently being processed is semantically connected with the available previously processed propositions. More precisely, propositions are connected via highly automated and non-strategic processes of memory resonance in search of previously processed antecedents to anaphoric references or to causal antecedents of, for example, story characters’ actions or emotions (Myers and O’Brien 1998; Singer et al. 1992). Currently processed information can resonate with information that has been received in the immediately preceding propositions, which means that local coherence is established. Currently processed information can also resonate with relevant information that has been received much earlier in the text and/or that refers to the overall topic, which means that global coherence is established (Albrecht and O’Brien 1993). The information necessary to form global coherence, in contrast to the information used to form local coherence, is retrieved from the text representation in long-term memory (Myers et al. 1994).

The plot of narrative texts is often structured by characters’ goals (Buss et al. 1983; Lynch and van den Broek 2007). Goals, therefore, serve as a useful framework for constructing a coherent mental representation by providing causal antecedents to characters’ actions and emotions (Bower et al. 1998; Singer 1992; Suh and Trabasso 1993; Graesser et al. 2001). This implies that comprehension of narrative texts often requires currently processed information to be linked with information about characters’ goals that does not necessarily still reside within working memory and thus relies not only on local but also on global coherence.

Connections on a global level, that is, connections with information that is not available within the working memory during the current processing cycle, seem to be routinely established, even when local connections, that is, connections with current working memory content, are possible (Huitema et al. 1993; Poynor and Morris 2003; Rizzella and O’Brien 1996). These findings are aligned with (Sanford et al.’s 1990) distinction between implicit and explicit focus (Myers et al. 1994): explicit focus refers to the currently processed information, whereas previously processed information remains in implicit focus. When processing narrative texts, the implicit focus may contain key characteristics of a protagonist, such as a goal. As soon as the protagonist is referenced and is, therefore, in explicit focus, an overlap with information stored in implicit focus occurs, which is reactivated and resonates with the information in explicit focus. Thus, the current reference to the protagonist is processed in accordance with the earlier introduced characterization and global coherence is established.

Overall, although highly automated, the process of constructing a locally and globally coherent mental representation of a text is a demanding process that requires working memory capacity (Just and Carpenter 1992), which develops greatly over the course of childhood and adolescence (Schneider 2015). The ability to construct coherence during text comprehension also develops during this time (Hacker 1997; Helder et al. 2016; Markman 1979; Oakhill et al. 2007; Vosniadou et al. 1988). Therefore, the question of how coherence formation can be supported in children is of great relevance. A promising approach is a text design that relieves the working memory and thus supports coherence formation and ultimately comprehension (see Graesser et al. 2001). This study examines illustrations, which are a common element of children’s books, as a possible mechanism to enhance local and global coherence formation.

### Processing of multimodal narratives

The combination of auditorily presented verbal information and illustrations creates a multimodal narrative in which the two modalities interact and contribute to an overall meaning (Cohn 2016). Comprehensive theories exist that attempt to explain how an integrated mental representation of text and pictures is constructed with reference to the human cognitive architecture and the nature of words and pictures as external representations (Mayer 2005; Schnotz and Mayer 2014). These theories stress two crucial aspects. First, they adopt the idea of dual channels within the working memory. They refer to Baddeley’s (1986, 2002) conception of the working memory with separate subsystems for the processing of phonological (written and auditory text) and visual-spatial information (pictures) as well as to Paivio’s (1986) dual coding theory which, besides also assuming different subsystems, emphasizes that verbal and non-verbal information are each stored in their own representational format. Thus, multimodal narratives make efficient use of the overall limited capacity of working memory: pictures added to verbal information, such as illustrations in children’s books, can be processed in parallel alongside the verbal information and provide additional memory cues (Fletcher et al. 2005). In addition, these theories emphasize that the information from both text and pictures is combined into an integrated mental representation of the text content.

Although these theories focus on the interplay of expository texts and charts, graphs, or schematic diagrams, their key assumptions seem to be applicable in the context of the current study. Previous research with narrative texts indicates that multimodality benefits children’s comprehension in general (see Carney and Levin (2002) for a review; Seger et al. 2019; Wannagat et al. 2018) and coherence formation in particular, but without differentiating between local and global coherence. Orrantia et al. (2014) examined 9- and 11-year-old children’s ability to establish global coherence when reading illustrated and unillustrated narratives. The reading times of sentences (that occurred in each the final section) that described actions that were inconsistent with a goal (described in each the beginning section) as well as the ability to detect these inconsistencies indicate that illustrations help in the process of connecting goals and actions. Similarly, Pike et al. (2010) found that pictures that illustrated key story aspects facilitated inferences that required later retrieval of this key aspect in 7- to 11-year-olds who read the stories.

### The current study

Overall, there is evidence that pictures added to verbal materials promote children’s text comprehension in general as well as processes of coherence formation. However, as discussed earlier, construction of coherence on both the local and global level is an essential requirement for text comprehension.

This study focused on children aged 9 to 12 years and examined the accessibility of causal antecedents to a protagonist’s emotion that were mentioned either in the beginning (indicating global coherence) or in the final sections (indicating local coherence) of narrative texts. We compared monomodal (auditory only) and multimodal (audiovisual) presentations of otherwise identical texts. We presented our materials auditorily to capture the coherence formation processes independently of the participants’ ability to decode letters (see Hoover and Gough 1990).

To operationalize these processes of local and global coherence formation, we adapted a procedure that was used previously by Rizzella and O’Brien (1996) and Unsöld (2008). They collected naming latencies of query words that were associated with a near or a distant causal antecedent of a character’s emotion or action. The rationale behind this procedure is that naming latencies depend on the accessibility of the associated concepts. Information necessary for local coherence formation (i.e., the near causal antecedents) is readily available within working memory. Information necessary for global coherence formation (the distant causal antecedents) has to be retrieved from long-term memory to be connected to a character’s emotion described in the last sentence of a narrative text in order to integrate this information into a coherent mental representation. To acknowledge the role of goals in narratives (Bower et al. 1998), in our materials, the distant causal antecedents were the story protagonists’ superordinate goals, which were described at the beginning of each text. The near causal antecedents were subordinate goals or obstacles that prevented the protagonists from achieving the superordinate goals; these were described in the final section of the text. The last sentence described the protagonists experiencing an emotion that could resonate with both the superordinate goal (distant antecedent) and the subordinate goal or the obstacle (near antecedent).

Because we presented the texts auditorily, we also presented the query words auditorily. Consequently, instead of naming latencies, we collected the response times to query words that were associated with distant and near causal antecedents in a memory probe task. Additionally, participants answered open-ended questions that required either an inference or a memory of a plot-relevant text detail. This was to ensure that the participants paid attention to the texts throughout the experiment and engaged in semantic processing.

The relation between text and pictures within our materials reflects the designs used in many illustrated children’s books. The narrative is guided by the verbally presented information, rendering the semantically dominant according to Cohn (2016). You can easily follow the narration based on the text without the pictures, but you cannot follow the narration based solely on the pictures. Rather, the illustrations enrich the verbally presented information; they provide the recipients with information about the appearance and surroundings of the protagonists and other characters that is not available to the same extent from the verbally presented information. Simultaneously, both the text and the sequence of pictures contain grammar; both make sense only in their respective designated orders. Overall, the relation between text and pictures can be described as verbal-assertive within Cohn’s (2016) cognitive framework for multimodal interactions.

In this study, we focused on the following hypotheses.

First, we assumed that accessibility of the near causal antecedents to the protagonists’ emotions (as indicators of local coherence) is higher than accessibility of the distant causal antecedents (as indicators of global coherence). The near causal antecedents should be readily available in working memory, while the distant causal antecedents have to be retrieved from long-term memory (Hypothesis 1).

Second, we assumed that accessibility of both the distant and the near causal antecedents is higher with a multimodal presentation (i.e., when pictures are present) than with a monomodal verbal presentation (Hypothesis 2). The illustrations that accompany the verbal narration can be processed in parallel alongside the verbal information due to the dual-structure architecture within the working memory (Baddeley 1986, 2002; Mayer 2005; Paivio 1986). Accordingly, the pictures might provide additional cues to resonate with the protagonists’ superordinate and subordinate goals (or obstacles), which serve as the distant and near causal antecedents to their emotion.

Third, we assumed that pictures increase accessibility of distant causal antecedents to a greater extent than accessibility of near causal antecedents. (Hypothesis 3). Accessing distant causal antecedents, which is information that is no longer available in the working memory, is the more demanding process. Thus, this process should benefit to a greater extent from the facilitating effect of pictures as additional memory cues.

## Method

### Participants

A sample of 114 children aged 9–12 years participated in the study. This corresponds to German grade levels 4–6. The materials were presented audiovisually to 59 children (29 boys, 30 girls; *M*_age_ = 10.5 years, *SD*_age_ = 1.1 years) and auditorily to 55 children (28 boys, 27 girls; *M*_age_ = 10.7 years, *SD*_age_ = 1.1 years). The mean age did not differ significantly between these two groups, *t *(112) =  − 0.914, *p* = 0.362.^1^

All participants either were native German speakers or had mastered German at a native-speaker level. We did not include children who had been diagnosed with attention deficit hyperactivity disorder and/or dyslexia as reported by parents or teachers. Only children whose parents had provided written informed consent were allowed to participate. The children were tested in schools during school hours.

### Memory probe task

For the purpose of this study, we developed 40 German narrative texts, which all had the same underlying structure, consisted of eight sentences each and described goal-based episodes (see Table 1 for two examples). As in real life, the protagonists in these texts sometimes attained their goal (texts) and sometimes they did not (texts). This variation was included to maintain suspense throughout the experiment and to reflect the reality of life in which you sometimes succeed and sometimes you do not, thus increasing the ecological validity of the experiment. A subset of these texts were also used in a revised form in the experiments described in Wannagat et al. (2020).

**Table 1:** The text on the left is an example of a story in which the protagonist reaches his goal. The text on the right is an example of a story in which the protagonists do not reach their goal

Goal reached	Goal not reached
Today is the last game of the season and Christoph tightly laces his soccer shoes. (Heute ist das letzte Spiel der Saison und Christoph schnürt seine Fußballschuhe fest zu.)	Lena and her older brother Finn are happy because their grandmother is visiting today. (Lena und ihr großer Bruder Finn freuen sich, denn die Oma kommt heute zu Besuch.)
He wants to perform at his best because the winning team will take home a nice trophy. (Er will sein Bestes geben, denn in diesem Jahr darf die Siegermannschaft sogar einen tollen Pokal mit nach Hause nehmen.)	At lunch time, Lena, Finn and their mother decide that they will cook mushrooms for dinner. (Mittags überlegen sich Lena, Finn und die Mutter, dass es zum Abendessen Pilze geben könnte.)
During the game, all players eagerly fight for the ball because they all want to get the trophy. (Während des Spiels kämpfen alle Spieler verbissen um den Ball, denn alle wollen unbedingt den Pokal holen.)	Lena and Finn suggest collecting the mushrooms in the woods behind their house. (Lena und Finn schlagen vor, die Pilze im Wald hinter ihrem Haus zu sammeln.)
Soon before the game is over, both teams are tied 1 to 1. (Kurz vor Spielende sind beide Mannschaften gleich auf und es steht 1:1.)	So, the three of them put on their jackets and cheerfully start walking. (So ziehen die drei sich ihre Jacken an und laufen frohen Mutes los.)
Christoph gets the ball and runs across the field. (Christoph bekommt den Ball und rennt über das Spielfeld.)	For a long time, they walk through rustling leaves, when suddenly dark clouds appear in the sky. (Lange laufen sie durch raschelndes Laub, als auf einmal dunkle Wolken aufziehen.)
Directly in front of the goal, he lunges and kicks the ball. (Kurz vor dem Tor holt er kräftig aus und schießt.)	They quickly hide under a big fir tree and heavy rain starts pouring down that just would not stop. (Schnell stellen sie sich unter eine große Tanne und prompt setzt starker Regen ein, der einfach nicht aufhören will.)
The ball hits the goal and then the referee immediately blows the final whistle. (Der Ball geht ins Tor und sofort danach pfeift der Schiedsrichter das Spiel ab.)	So, the mother takes Lena’s hand and the three of them start running home through the rain as fast as they can. (So nimmt die Mutter Lena an die Hand und die drei rennen so schnell sie können durch den Regen zurück nach Hause.)
Christoph smiles happily. (Christoph strahlt über das ganze Gesicht.)	Back home, they are in a very bad mood. (Im Haus angekommen sind alle drei ziemlich schlecht gelaunt.)

The first sentence introduced the general setting of the narrative. In the second and third sentences, a (superordinate) goal was described. In the example text about Christoph, this goal was to win a trophy (Table 1, left column). In the example text about Lena and Finn, the goal was to collect mushrooms (Table 1, right column). The fourth and fifth sentences had the purpose of advancing the plot without explicit reference to the earlier described goal. Then, sentences six and seven introduced either a subordinate goal that helped the protagonist reach his or her superordinate goal or an obstacle that prevented the protagonist from reaching the previously described superordinate goal. In the example story about Christoph, he scores the decisive goal that causes his team win the trophy. In the story about Lena and Finn, heavy rain sets in that prevents them from continuing their efforts of finding mushrooms. Finally, the last sentence described an emotion that the protagonist(s) experience(s). To coherently integrate the last sentence with previously processed information, it has to be connected to a causal antecedent. The text provides two possibilities. On the one hand, the last sentence can resonate with the distant causal antecedent, which is the superordinate goal that has been described in the second and third sentences of the story. This would constitute a global connection. For the example texts, this would mean that Christoph is happy because he wins the trophy and that Lena and Finn are unhappy because they could not find any mushrooms. On the other hand, the last sentence can resonate the near causal antecedent, which is the subordinate goal or the obstacle that has been described in the sixth and seventh sentences. This would constitute a local connection. For the example texts, this would mean that Christoph is happy because he scored a goal and that Lena and Finn are unhappy because it started raining. We recorded the texts with a female German native speaker at the department’s recording studio. The mean text duration was 38.88 s (*SD* = 5.92 s).

For the audiovisual versions, we additionally created five colored pictures, which were presented alongside and for the duration of the recording of the respective sentences. Figure 3 shows the pictures that illustrated the example texts that are displayed in Table 1. While not every sentence was illustrated with its own picture, for every text, the first and last sentences and at least every second sentence were illustrated. In addition, sentences that described near and distant causal antecedents were each illustrated equally often across all texts. The pictures were illustrations of the situations described in the corresponding sentences. This means that the pictures depicted the protagonists (in 88 out of 100 pictures), other story characters (10 pictures) or objects that are important for the plot (2 pictures). The picture that illustrated the final sentence that described the protagonists’ emotion was always a close-up of the protagonists’ face(s). When no picture was presented along with the sentence, a blank (white) screen was shown instead.

**Fig. 1:**
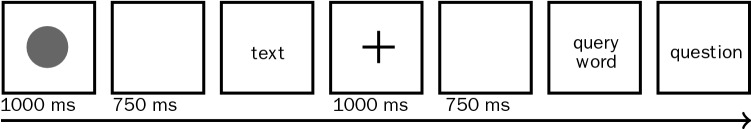
Sequence of events in an example trial. The dot was blue. Note that a question was asked after each 20 of the 40 total texts

During the experiment, these texts were presented either auditorily (55 participants) or audiovisually (59) on a computer screen, one after another, resulting in 40 trials (see Fig. 1 for a detailed depiction of the procedure). After every text, the participants were presented with a query word (auditorily). It was their task to decide, as quickly and as accurately as possible, whether this word had occurred in the text. They entered their answer using two specially prepared keys, one marked with a black cross against a red background (“no”) and the other marked with a black tick against a green background (“yes”).

In addition to the query words, immediately following each 20 of the texts, the participants were asked to answer a comprehension question that required either naming a plot-relevant text detail or making a plot-relevant inference. Recordings of these questions were played automatically after the answer regarding the query word had been entered. Children answered the experimenter orally, who noted the answers on a protocol sheet. For example, the question about the text about Christoph was “Which team wins?”.

The query word was either a so-called global query word that was associated with the superordinate goal (10 texts) or a so-called local query word that was associated with the subordinate goal or the obstacle (10 texts). These query words had been mentioned twice in the respective sentences that described these goals or obstacles (see the words printed in italics in Table 1; trophy/goal and mushrooms/rain). The query words were not shown in the pictures. This was to rule out the possibility that the audiovisual presentation would be beneficial simply because the query words were presented twice (auditorily and as a picture).

**Fig. 2:**
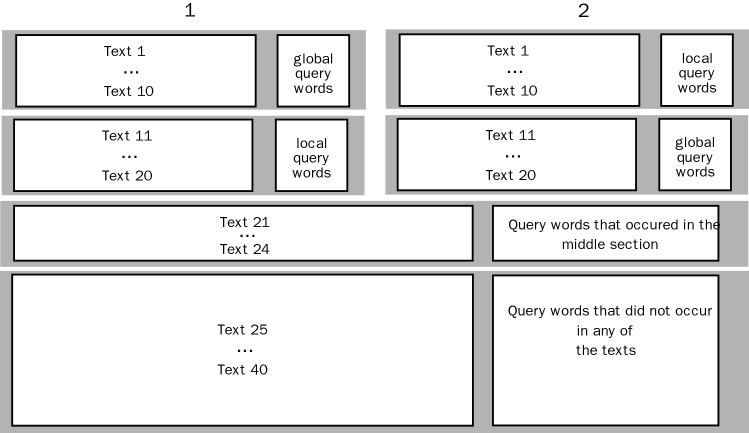
Balancing of texts and query words. Texts 1–20 are the experimental texts, while texts 21–40 are the filler texts. In texts 1–10 and 21–30, the protagonists achieved their goals, and in texts 11–20 and 31–40 they did not. To ensure that across all participants every experimental text was presented followed by each its local and its global query word equally often we programmed two conditions (1, shown on the left, and 2, shown on the right). Conditions 1 and 2 were alternated between participants. The order of presentation of the overall 40 texts was determined randomly by the experimental software for every participant. Questions were asked about texts 2, 4, 8, 9, 11, 15, 17, 18, 19, 22, 23, 24, 25, 28, 29, 30, 31, 32, 35, and 40

Twenty of the texts served as filler texts. These texts were either followed by a query word that had appeared in the middle of the respective text (4 texts) or by a query word that had not appeared in any of the texts (texts). Thus, the overall ratio of required yes and no answers was 60:40. The query words from the middle sections served the purpose of preventing the participants from directing their attention only to the first and last sections, in which the local and global query words appeared. For every participant, the order in which the texts were presented (20 experimental texts and 20 filler texts) was determined randomly. We used PsychoPy. (Peirce 2009) to program the experiment. Across all participants, every text was presented equally often in combination with its local and its global query word (see Fig. 2 for a detailed description of the balancing).

**Fig. 3:**
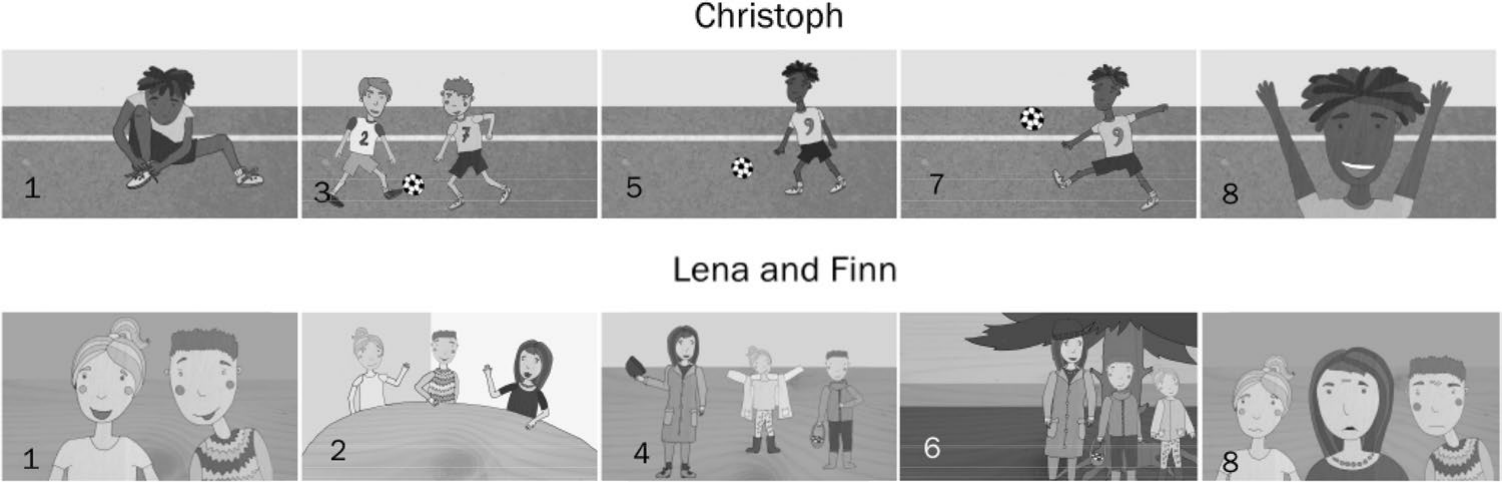
The pictures illustrating the example texts about Christoph (Table 1, left column) and Lena and Finn (Table 1, right column). The numbers in the bottom left corners correspond to the sentence the respective picture illustrates. Colored versions of these pictures were used in the experiment

The experiment took approximately 45 min. Every participant was tested individually. Before the actual experiment started, participants listened to two practice texts—one with a query word that had occurred in the text and another in which the query word had not occurred. In these practice texts, participants received automated feedback about the correctness of their answers.

We compared the response times between auditory and audiovisual presentation separately for each type of query word (global and local). Faster responses to global query words in one presentation mode would indicate a higher accessibility of the distant causal antecedents in this mode of presentation compared to the other. Accordingly, faster responses to local query words in one presentation mode would indicate a higher accessibility of the near causal antecedents (subordinate goals or obstacles) in this mode of presentation compared to the other.

#### Pilot study and preliminary analyses

We conducted a pilot study to validate the association of the global and local query words with the superordinate and subordinate goals and the obstacles. Ten students read the texts and were asked to name two reasons that the protagonist(s) was experiencing the emotion described in the last sentence (in the example text: “Why did Christoph smile happily?” Please name two reasons). Depending on the answers, we revised the query words in some cases. In addition, by collecting independent ratings, we ensured that the things or events that constituted the superordinate and subordinate goals were equally desirable in the context of the stories. In the case of stories in which the goals were not achieved, we collected ratings on whether the confrontation with the obstacle was annoying to a comparable degree as reaching the superordinate goal was enjoyable.

The mean duration of the audio recordings was 617.350 ms (*SD* = 141.572 ms) for the local words and 613.200 ms (*SD* = 135.857 ms) for the global query words and did not differ statistically from each other, *t *(38) = 0.095, *p* = 0.925. Thus, it is highly unlikely that response time differences between local and global query words can be attributed to word length.

Another variable that might affect response times is the participants’ familiarity with the words used as local and global query words. Therefore, we looked up the word frequencies (log-10 annotated type frequencies) in the childLex database (Schroeder et al. 2015). This database contains a corpus of 10 million words used in German children’s books and offers linguistic parameters for children aged between 6 and 12 years. The mean log-10 annotated type frequency did not differ statistically between the local and global query words, *t *(38) = 1.232, *p* = 0.225. However, the descriptive statistics suggested that the words we used as global query words (*M* = 74.480, *SD* = 96.044) are more frequent than the words that we used as local query words (*M* = 44.965, *SD* = 50.663). We discuss the implications of this discrepancy later.

### Data preparation and analysis

Before the data analysis, we first excluded data points when the answer was inaccurate. The error rate regarding the memory of global query words was 7.719% (9.455% in the auditory condition and 6.102% in the audiovisual condition), while that regarding the memory of local query words was 1.404% (1.818% in the auditory and 1.017% in the audiovisual condition).

Second, to eliminate outliers, we removed response times greater than 5,000 ms. Then, we log-transformed the response times and subsequently eliminated data points that lay two standard deviations above or below the mean within the respective subsample (presentation mode and query word). Ultimately, 90.921% of the data points (response times) were included in the analyses.

To analyze the response times, we fitted a linear mixed model using the lmer function (package lme4; (Bates et al. 2015) in R, version 3.5.1, with fixed effects of presentation mode (auditory vs. audiovisual, contrast coded) and query word (local vs global, contrast coded). Because of the wide age range within the sample, we also included a fixed effect of age (*z*-standardized values of the age in months). We analyzed interactions using the emmeans function of the emmeans package (Lenth 2018). We further included random intercepts for the participant and item levels to account for the intraclass correlations for participants (ICC = 0.378) and items (ICC = 0.083) in the respective unconditional models. A model with random slopes for presentation mode or query word did not converge.

For every correct answer to an open-ended question, we awarded one point. Half a point was awarded for partially correct answers. Two members of the research team independently rated the answers and discrepancies were resolved by discussion. Thus, a maximum of 20 points could be achieved.

## Results

### Analysis of the comprehension questions

The participants answered open-ended questions after each 20 of the overall 40 texts. Due to the random order of text presentation, it was impossible to anticipate which text would be followed by a question. The mean score was 17.310 points (*SD* = 2.073) when texts had been presented audiovisually and 15.787 points (*SD* = 2.760) when texts had been presented auditorily. Although the performance was significantly better after audiovisually presented texts, *t *(110) =  − 3,317, *p* = 0.001, we conclude that the performance in both modalities indicates that the participants constantly payed attention to the text content and thus presumptively engaged in semantic processing of the information presented to them during the experiment.

### Analysis of response times

Table 2 displays the estimates of the fixed effects for the linear mixed model of the log-transformed response times.

**Table 2:** Estimated coefficients of the fixed effects, standard errors (SE), degrees of freedom (*df*) and *t*-values for the linear mixed model for the log-transformed response times

	Estimate	SE	*df*	*t*	*p*
(Intercept)	7.265	2.515	1.281	288.909	< 0.001
Age	− 6.256	1.584	1.338	− 3.949	< 0.001
Query word	1.086	2.417	4.836	4.494	< 0.001
Presentation mode	− 7.645	2.800	1.342	− 2.730	0.007
Query Word × Presentation mode	− 4.120	1.605	1.940	− 2.567	0.010
Age × Presentation mode	2.042	2.186	1.375	0.934	
Age × Query word	6.431	9.260	1.930	0.694	
Age × Presentation Mode × Query word	− 5.854	1.276	1.956	− 0.459	

*Note* Query Word (contrast coded: global = 1, local =  − 1), Presentation Mode (contrast coded: audiovisual = 1, auditory =  − 1)

This analysis revealed a significant effect of age (*p* < 0.001) but no interaction of age with any of the other predictors. The older the participants, the faster they responded, irrespective of the query word and the presentation mode. This finding replicates the well-known increase in general processing speed during the course of childhood (Kail 2000). Therefore, we do not interpret this finding further with reference to age effects in terms of the accessibility of causal antecedents.

Table 3 displays the means (and standard errors) of the response times, as estimated based on the linear mixed model displayed in Table 2.

**Table 3:** Mean (*M*) response times (and standard errors, SE) to global query words (RT_global_) and local query words (RT_local_) in the auditory and the audiovisual conditions as well as overall values

	RT_global_ (ms)	RT_local_ (ms)	RT_overall_ (ms)
	*M (SE)*	*M (SE)*	*M (SE)*
Auditory	1587 (37)	1436 (34)	1510 (34)
Audiovisual	1422 (40)	1326 (37)	1366 (30)
Overall	1495 (31)	1380 (29)	

*Note: *The reported values are back-transformed values of the values estimated within the model reported in Table 2

In line with our first hypothesis that near causal antecedents would be more easily accessible than distant causal antecedents, we found a significant main effect of the factor query word (*p* < 0.001). The responses to local query words (i.e., the query words associated with the subordinate goals that constitute the near causal antecedents in the experimental texts) were faster than those to global query words (1380 ms vs. 1495 ms).

Furthermore, we assumed that an audiovisual presentation would facilitate accessibility of both near and distant causal antecedents (Hypothesis 2). This assumption is reflected in the significant main effect of the factor presentation mode (*p* = 0.007), with generally faster responses when the texts had been presented audiovisually vs. auditorily (1366 ms vs. 1510 ms).

Finally, we assumed that the beneficial effect of the audiovisual (vs. auditory) presentation would be greater with regard to the accessibility of distant compared to near causal antecedents (Hypothesis 3). In line with this assumption, we found an ordinal interaction of Query Word × Presentation Mode (*p* = 0.010). Subsequent Tukey-adjusted contrasts revealed that response times to both local, *t *(140) = 2.691, *p* = 0.008 and global query words, *t *(143) = 4.115, *p* < 0.001, were faster when texts had been presented audiovisually (vs. auditorily), but the difference between the auditory and audiovisual presentations was larger for global query words (165 ms) than for local query words (110 ms).

When adding goal attainment to the model as an additional fixed effect on an exploratory basis, the model fit, based on the AIC, did not improve compared to the reported model (shown in Table 2), χ^2^ = 10.013, *p* = 0.264. This indicates that this additional layer of complexity does not seem to add substantial information.

#### Summary of the results

The response times to local query words were faster than those to the global query words. Additionally, we found faster responses to both global and local query words when the texts were presented audiovisually as compared to an auditory presentation. This effect was slightly larger with respect to global query words.

## Discussion

The purpose of this study was to examine the effect of illustrations on children’s comprehension of narrative texts. We compared, in children aged 9 to 12 years, local and global coherence formation as cognitive processes that are fundamental for text comprehension between monomodal (auditorily presented verbal-only) and multimodal (audiovisual) narrative texts.

For this purpose, we analyzed the response times of 114 children who completed a memory probe task. The participating children listened to a series of auditorily or audiovisually presented goal-based narrative texts. The protagonist’s superordinate goal, which was described in the first part of each text, constituted a distant causal antecedent to the protagonist’s emotion described in the last sentence. The protagonist’s subordinate goal or an obstacle, which was described in the final part of each text, constituted a near causal antecedent. The memory probe task that followed each text required the participants to answer, as quickly as possible, whether a query word associated with either the distant or the near causal antecedent had occurred in the respective text.

Establishing coherence—that is, in the context of the current study, causally connecting currently processed text information with a suitable antecedent—requires the causal antecedent to resonate with information that is currently active in memory (Myers and O’Brien 1998). Therefore, we used the response times to query words, which reflected access to, or memory retrieval of, the associated concepts (the distant and near causal antecedents), as indicators of global and local coherence formation processes.

By definition, a near causal antecedent is presented in close spatial and temporal proximity to its consequence within a text. Therefore, in comparison to a more distant causal antecedent (represented in the current study by the story protagonists’ superordinate goals), a near causal antecedent (the story protagonists’ subordinate goals or obstacles) can be assumed to be available within working memory. It should be more easily accessible than a distant causal antecedent, which has to be retrieved and reactivated from the text representation in long-term memory (Myers et al. 1994). In short, we assumed that establishing local coherence should be easier than establishing global coherence (Hypothesis 1). In line with this assumption, we found faster mean response times to query words associated with the near causal antecedents (local query words) compared to their responses to query words associated with the distant causal antecedents (global query words).

### Comparison between auditory and audiovisual text presentation

The primary purpose of this study was to compare monomodal (auditory verbal information) with multimodal (audiovisual) text presentation in terms of processes of local and global coherence formation in children aged 9–12 years. In the multimodal presentation, the verbal information guided the narrative and was accompanied by illustrating pictures. Thus, the illustrations enriched the verbal information and were associated with the protagonists’ goals without explicitly depicting them. We expected the information shown in the pictures to serve as cues to the protagonists’ superordinate and subordinate goals, which would resonate as causal antecedents to the emotion described in the last sentence of the narratives. Thus, accessibility of both the near and the distant causal antecedents should be facilitated by accompanying pictures (Hypothesis 2). This hypothesis was based on the notion that the information conveyed in the pictures, and the verbal information are processed simultaneously in distinct channels within the working memory (see Mayer 2005). Thus, during every processing cycle (see Kintsch 1988), information from both the pictures and the verbal information is available, creating an integrated and more comprehensive mental representation that provides a wealth of cues for local and global coherence formation. Overall, a multimodal presentation makes efficient use of working memory, which is of particular relevance for children because their working memory capacity is not yet fully developed (Schneider 2015).

Consistently, we found faster responses in children aged 9 to 12 years when texts were presented audiovisually, irrespective of the query word. Analyses separated by query word revealed this effect for both local and global query words. This result leads to two conclusions. First, in line with our assumption, illustrations seem to facilitate memory resonance with both near and distant antecedents, thus facilitating accessibility of both near and distant causal antecedents. This expands the previously referenced findings of Orrantia et al. (2014) and Pike et al. (2010) that indicated that illustrations facilitate the ability of children aged 7 to 11 years to connect distant text elements with each other. Second, this result indicates 1) that this effect is obtained even when the illustrations are parsimonious as, in our materials, only five out of eight sentences were illustrated and 2) that the illustrations do not need to explicitly depict the coherence-relevant information.

Furthermore, we assumed that accessibility of distant causal antecedents would be facilitated to a greater extent than that of near causal antecedents (Hypothesis 3). Both processes depend on memory resonance with a relevant antecedent, but for local coherence, the information needed is immediately available in the working memory. Therefore, this process might not depend on additional cues as much as global coherence formation does as it requires the currently processed information to be connected to the text representation in the long-term memory. In line with this assumption, we found an ordinal interaction between the predictors query word and presentation mode. This interaction indicates that the difference in the mean response times between auditory and audiovisual presentations was slightly larger when considering global query words (165 ms) compared to local query words (110 ms). This finding allows the tentative conclusion that the benefit of adding pictures to verbal information is larger in the context of the more demanding task of connecting distant text information. Of course, the discrepancy in the response times is not very large and more research is needed to explore the practical significance of this effect.

### Limitations and directions for future research

One may argue that it is possible to decide based on text surface memory whether a query word that appeared in a text in its verbatim form had appeared in the text preceding this task. For the response times to be a valid measure of the cognitive processes necessary to establish local and global coherence, the study participants were required to engage in semantic processing. To encourage them to do so, we asked the participants to give the answer to an open-ended comprehension question to the experimenter after each 20 of the texts. Because of the random order of text presentation, the participants could not predict after which of the texts a question would be asked. Furthermore, to prevent them from directing their attention especially to the first and final sections of the texts, in some of the filler texts, the query word was one that had appeared in the middle section. The mean score achieved by answering the comprehension questions, in our opinion, indicates that the study participants engaged in semantic processing of the text content in both the auditory and the audiovisual condition. The content of the illustrations provides further support for this interpretation of the response times. The query words were not part of any of the illustrations. As discussed, this implies that the difference between auditory and audiovisual presentation cannot simply be explained by the fact that the explicitly task-relevant information (i.e., the query words) were presented twice, once as a word and once as a picture. This further motivates our interpretation of the response times as indicating processes of memory resonance and thus of semantic processing beyond text surface memory. Future research may further clarify this issue by using query words that are implied but not explicitly mentioned (see also Wannagat et al. 2020).

Another presumed limitation concerns the fact that the local and global query words were different words. As stated in the method section (see p. 10), in addition to their function as local and global query words, the participants’ familiarity with the words presumably affected how easily and quickly they were recognized. We consulted the childLex database (Schroeder et al. 2015) for word frequencies as indicators of the participants’ familiarity with the words that served as query words. When considering a descriptive comparison, the global query words turned out to be more frequent than the local query words (*M* = 74.480, *SD* = 96.044 vs. *M* = 44.965, *SD* = 50.663; log-10 annotated type frequency).^2^ Drawing on this observation, the global query words should have been recognized more easily and, thus, more quickly. Instead, as predicted from a theoretical point of view, the response times were faster for local than for global query words in both experiments. This means that, if anything, the effects concerning the differences between global and local query words are under- and not overestimated. Therefore, the differences between query words in terms of mean word frequencies do not seem to restrict the implications of the findings reported in this paper (see also Wannagat et al. 2020).

Besides the previously discussed main findings, this study revealed better performance for the questions that asked for plot-relevant details or information that needed to be inferred based on the text content when texts were presented audiovisually (vs. auditorily). Notably, these questions were included to ensure that the participants paid attention to and engaged in semantic processing. This secondary finding indicates that the beneficial effect of audiovisual over auditory presentation is not specific to coherence formation. Audiovisual presentation also seems to support other cognitive processes relevant for text comprehension. This notion aligns well with previous reviews on the effect of pictures on children’s text comprehension (see e.g., Carney and Levin 2002). Future research may investigate the effect of pictures on possible interactions of different cognitive processes by including a broader variety of outcome measures as well as person-level predictors, such as motivation, engagement or attention. Another promising starting point would involve exploring whether the nature of the text–picture relation proposed by Cohn (2016) moderates the extent to which pictures support the establishment of coherence. Overall, additional research is needed to further shed light on how, why, to what extent and under what circumstances pictures become effective.

### Conclusion

In conclusion, this study extends existing findings describing the beneficial effects of multimodality over monomodality on children’s narrative text comprehension and coherence formation. It is the prevailing view that text comprehension requires a recipient to establish both local and global coherence and our findings suggests that pictures added to auditory text have a beneficial effect on both processes.

## Footnotes

1 Originally, 120 children signed up for participation. Before each day of testing, it was decided randomly which of the children would be presented the materials audiovisually (60 overall) and which, auditorily (60 overall). One child who had been assigned to the audiovisual group did not attend school on the day of testing. Five children assigned to the auditory condition did not attend school on the day of testing or did not complete the experiment.

2 Note that the descriptively large differences in mean frequency between local and global query words and the particularly large standard deviation of the frequency of the global query words can be attributed to a great extent to the word “Schule” (school). This word has a disproportionately high value. An analysis of the data that excluded this item did not yield different results.
